# The effects of training and creatine malate supplementation during preparation period on physical capacity and special fitness in judo contestants

**DOI:** 10.1186/1550-2783-9-41

**Published:** 2012-09-03

**Authors:** Stanislaw Sterkowicz, Anna K Tyka, Michal Chwastowski, Katarzyna Sterkowicz-Przybycień, Aleksander Tyka, Artur Klys

**Affiliations:** 1 Department of Theory and Methodology of Combat Sports, University School of Physical Education, Al. Jana Pawla II 78, 31-571, Cracow, Poland; 2 Department of Recreation and Biological Regeneration, University School of Physical Education, Al. Jana Pawla II 78, 31-571, Cracow, Poland; 3 Doctoral Studies, University School of Physical Education, Al. Jana Pawla II 78, 31-571, Cracow, Poland; 4 Department of Theory and Methodology of Gymnastics, University School of Physical Education, Al. Jana Pawla II 78, 31-571, Cracow, Poland; 5 Institute of Human Physiology, University School of Physical Education, Al. Jana Pawla II 78, 31-571, Cracow, Poland

## Background

The supplementation of standard diets with creatine-based compounds in speed-and-strength sports has become very popular today. The creatine alone is an endogenous substance synthetized in internal organs, such as liver, pancreas and kidneys. Primary stores of free creatine (Cr) and its phosphorylated form (PCr) are skeletal muscles, cardiac muscle and smooth muscle tissues. Since the mechanism of phosphocreatine shuttle was described in 1981, the role of this compound in cellular metabolism has increased dramatically [1,2]. In athletes competing in speed and strength sports, such as combat sports, particularly in judo, the demand for ATP is elevated during the physical exercise of interval character. An energy substrate required for its re-synthesis is phosphocreatine which, combined with ADP and creatine kinase (CK), synthesizes ATP [3,4]. The efficiency of these processes might have a significant effect on the effectiveness of a judo fight.

Supplementation of diets for athletes from a variety of sports with creatine-based compounds is associated with an improvement in physical performance of speed and strength character. Previous studies have shown that supplementation of diets with creatine positively affects physical performance in terms of the ability to generate peak power and the power in repeated anaerobic exercise [4-6]. Legal substances used so far, with the efficiency that has been determined empirically, include creatine monohydrate citrate, creatine malate and creatine ester. The use of creatine malate for tests carried out among judoists in the present study was not accidental as it resulted from the lack of empirical data in the available scientific literature and the necessity of determination of its actual effect on physical capacity in judoists. Few studies have examined this substance in groups of track and field athletes, mainly sprinters and long distance runners, and have demonstrated its ergogenic effect only in sprinters [4]. Increased fat-free mass (FFM) during anaerobic test was accompanied by elevated absolute and relative results concerning peak power (PP) and total work (TW). Although the creatine malate, which is a compound of three particles of creatine connected, through an ester bond, with one particle of malate, has two weak bonds which are susceptible to esterase, its one strong bond is secure enough to prevent the creatine particle from its conversion into creatinine. In this form, the creatine absorption and digestion is much more efficient compared to other preparations [4]. Creatine malate was chosen as a suplement for its vital role in generating muscle power [7]. What is more creatine malate supplementation comparing to monohydrate helps to avoid accumulating water in muscle cells [8] as well as it is easierly absorbed from the digestive system, which coincides with better solubility in water.

Although judo is a sport which is complex, both technically and tactically, the expectations of post-exercise changes in physical capacity during non-specific laboratory tests seem to be justified. “Under competitive conditions, with intermittent character of exercise, where ratio of intensive exercise bouts during the fight to rest time typically amounts to 2:1 [9], the training process require a fine integration of aerobic and anaerobic training [10].

Therefore, it seems reasonable to formulate a hypothesis of the effect of training on the improvement in results obtained during a specific intermittent test, i.e. the SJFT test [11]. The hypothesis concerning the changes in physical capacity and special fitness in athletes who supplement diets with creatine compounds also seems interesting.

The main aim of the present study was to carry out observations and analyze changes in body composition, anaerobic and aerobic power and special judo fitness induced by six weeks of judo training. An additional aim was to examine the effect of supplementation with creatine malate on body composition and physical capacity indices and special fitness of judoists.

## Methods

### Subjects

Age of the male subjects (n = 10) who took part in the study ranged from 17 to 28 years with the average of 21.2±3.3 years, whereas their training experience ranged from 5 to 21 years (11±4.5 years). Three of them had 2^nd^ Kyu judo rank, three of the subjects had 1^st^ Kyu and four of them had 1^st^ Dan level.

### Procedures

Body height, measured by means of Martin’s anthropometer, varied from 1.68 to 1.87 m (1.75±0.06 m). Measurements of body mass (BM) were taken with the accuracy ±1g by means of F1505-DZA Sartorius (Germany) scales. Measurements were carried out according to the recommendations in kinanthropometry [12]. The three consecutive measurements of two skinfolds (triceps and subscapular) were taken with GPM skinfold caliper with measurement range 0–45 mm ± 0.2 mm, made in Switzerland, further on intra-observer error was counted. Typical error of skinfolds measurement [13] were 1.8% for triceps, and 2.0% for subscapular. The both measurement were within proper anthropometric tolerance (5%), which is recommended for skinfolds measurements [12]. Intraclass correlation coefficients 3,1 (ICC > 0.95) show high reliability of repeated skinfolds measurements [13,14]. Percent fat (PF) was estimated by means of the formula for white postpubescent and adult males [15], which takes into consideration the thickness of triceps skinfolds and the inferior angle of scapula. BMI and body composition indices, such as fat-free mass index (FFMI) and fat mass index (FMI) were calculated [16]. Biometrical measurements, Wingate tests for anaerobic capacity (ICC for relative peak power was 0.85 and typical error = 0.31 W·kg^-1^) and graded exercise for aerobic power were repeated twice in the athletes who were during preparation period. The subjects also performed the special judo fitness test, SJFT [11]. The time interval between the measurements 1 and 2 amounted to 6 weeks.

#### Characteristics of the training aimed at preparation for the second half of the annual training cycle

The contestants have been training for approximately 20 hours a week: 5 days for 2 two-hour-training session. During the first stage, in the beginning of the preparation period, the contestants participated in a two-week training camp which was aimed at base training before special judo training regimes and competitive seasons were started. The physical exercise was aimed at the development of endurance by means of continuous training and interval methods in the form of running and rowing. Strength training was dominated by the exercises with partners which were based on repetitions. The contestants also practiced the fight in standing position (Tachi-waza) and ground fighting (Ne-waza), which helped to solve a variety of tactical problems. Two training sessions took place every day.

During the second stage of the preparation, the subjects participated in a 4-day camp which involved fighting (Randori) twice a day. Three days later, a similar week-long camp was organized in Slovenia, where the judoists practiced sparing fights on the judo mats. The aim of this training procedure was to improve endurance and special strength and development of technical and tactical skills of sport fighting.

Next two weeks involved training with the character of direct preparation for competition and it was oriented towards the development of speed and speed endurance. Directly after this period the second testing was performed. After next five days, 7 of 10 contestants participated in the international tournament in Banska Bystrica. Five of them scored places from 1^st^ to 5^th^ in their weight categories (this event is not presented in the international ranking system of International Judo Federation).

#### Characteristics of supplementation procedure

A randomly selected study group (n = 5) was subjected to 6-week supplementation with creatine malate (“TCM”, Olimp Labs, Poland). There are different approaches for calculating the amount of creatine malate, one is that endogenic creatine for a person whose weight is 70 kg should be 2 g [17], which are lost during the day and half of this amount is synthetised in the liver. This is why 1 g should be delivered with food [18]. The optimal amount of creatine malate used for supplementation was calculated with formula 0.07 g^.^kg^-1^LBM which corresponded to 5 g of creatine malate for a person with LBM of 70 kg [19].

Every day, two hours before the breakfast, the capsules containing 0.07 g^.^kg^-1^ LBM of the preparation were administered orally, which corresponded to ca. 5 g for a person with FFM of 70 kg [19]. The supplement was dosed with 250 ml of clean, room temperature water. Other subjects were given placebo in similar capsules. The judoists did not ingest other supplements during the study. After the loading phase, the final examinations were carried out in order to determine the effect of training and supplementation on judo contestants. No statistically significant differences in age (T = 20.4±3.0, Me = 20 vs. C = 22.0±3.7, Me = 22 years, P > 0.05), training experience (T = 11.0±6.0, Me = 10 vs. C = 11±3.0, Me = 10 years, P > 0.05), and sports achievements were noticed. Judoists took part in both national and international contests. In both groups (T and C) one of the competitors was ranked in International Judo Federation. Double-blind placebo controlled design have been used.

### Anaerobic test

The subject started the proper test after 5 minutes of warm-up with the intensity of 50% of VO_2_max using bicycle ergometer with pedaling rate of 70 revolutions per minute and three maximum acceleration bouts in 5 last seconds of the 2nd, 4th, and 5th minute and after 2-minute rest after the completion of this warm-up. The anaerobic test was a modified form of the Wingate test [20]. The load on the ergometer platters, which was optimum for each athlete, was determined during a pilot study and amounted to 8.3% of BM on average, i.e. by 0.8 higher than in the original. With this braking force, the athletes generated the greatest peak power. It consisted in pedaling for 30 seconds with maximal intensity using a mechanical bicycle ergometer Ergomedic 874E manufactured by Monark.

During the exercise, a computer recorded relative peak power (RPP) and relative total work (RTW), time to obtain peak power (toPP), time to maintain peak power (tuPP) and the fatigue index (FI).

### Graded test until fatigue

A graded exercise test on a mechanical treadmill was carried out on the second day of the experiment, under similar ambient conditions. After the determination of pre-exercise circulatory and respiratory indices, the subjects performed a 3-minute warm-up at the running speed of 2.3 m^.^s^-1^, and then the speed was increased by 0.5 m^.^s^-1^ every three minutes. During the last 30 seconds of each loading segment, the subjects were taken blood samples from the earlobe in order to determine the lactate concentration in blood serum. The graded exercise was continued by the subjects until a subjective sensation of exhaustion. Using the apparatus of 919E type manufactured by Medikro, the indices of respiratory exchange were measured during the exercise every 30 seconds: tidal volume (TV), respiratory rate (F), minute ventilation (V_E_), minute oxygen uptake (VO_2_). Heart rate monitor Vantage^TM^ manufactured by Polar Electro was used for the measurements of heart rate (HR). Total time of exercise (t) and the distance (D) were also recorded. It was established based on a pilot study that the capillary blood samples used for the determination of biochemical and morphological indices would be also taken from the earlobe three minutes after the exercise. This was the point when the highest lactate (La) concentration was found. All the exercise tests were performed in an air-conditioned room in the Department of Physiology and Biochemistry of the Institute of Human Physiology. The project was approved by the Bioethical Committee at the Regional Medical Chamber (No. 76KBL/OIL/2008 of 17 September 2008).

### Special Judo Fitness Test

Special Judo Fitness Test (SJFT) invented by one of the authors of the present study is an acknowledged tool of training control, implemented in many countries [11]. Visualized presentation was prepared at the University of Bath by Lance Wicks [21]. The test positively passed the statistic procedures determining the reliability and accuracy, and had normative data [11]. SJFT is a recognized tool used also in judo-related disciplines, such as ju-jitsu, hapkido etc.

### Statistical analysis

The following descriptive statistics were calculated: mean, SD, median. Non-parametric methods were used, because not all parameters show normal distribution. Based on the differences between results of measurement 1 and measurement 2, the statistical significance of changes was evaluated for individual indices of body build and composition, physical capacity and fitness by means of Wilcoxon matched pairs test. The assessment of the effect of supplementation on dependent variables was based on the Mann–Whitney U test. The post-test measurement (measurement 2) is the response of the two groups (T group [n = 5]: supplementation with creatine malate; C group [n = 5]: placebo). These groups were not differed according to age, sport experience and competitive level (national and international level were presented by 4 and 1 competitors in each group). The comparisons were focused on relative data values and indices. Statistical hypotheses concerning the differences between the medians were verified at the level of significance of P < 0.05.

## Results

The initial level of body mass in the contestants ranged from 61.2 to 101.2 kg (76.09 ± 14.85 kg, Me = 70.73 kg) and was lower (z = 2.40, P < 0.05) than in the second test, when it ranged from 63 to 102.9 kg (78.52 ± 14.53 kg, Me = 75.30 kg). The significant difference (z = 2.30, P < 0.05) was observed in FM and FMI, but not in percent fat in body mass (PF%). FM and FMI contributed in increased body mass and BMI (z = 2.20, P < 0.05) (see Table 1). Tables 2 and 3 present changes occurring in anaerobic capacity and aerobic power before and after the six-week training during preparation season. A significant difference (z = 2.09, P < 0.05) in the level of toPP points to advantageous shortening of the time needed to generate peak power (Table 2). The index of aerobic power in measurement 2 exhibited a decrease compared to the measurement 1, but the differences were not significant (P > 0.05). In both measurements of VO_2_max higher results were observed in T comparing to C group). Percent at VO_2_max at the anaerobic threshold (%VO_2_max), in the first measurement showed no significant differences between two groups, while in the second measurement statistically significant differences were observed: in T group %VO_2_max was higher (Table 3).

**Table 1:** Body build and body composition changes in judoists during preparation period (mean ± SD, Median)

	**Pre**	**Post**
BMI (kg·m^-2^)	24.59 ± 3.41; 22.99	25.32 ± 3.34; 24.93^#^
C	22.27 ± 0.97; 22.85	23.26 ± 1.80; 23.04
T	26.92 ± 3.41; 27.93	27.38 ± 3.36; 28.09
FFM (kg)	68.44 ± 12.81; 63.08	70.05 ± 12.72; 64.33
C	59.96 ± 5.07; 60.07*	62.36 ± 5.68; 59.89
T	76.91 ± 12.80; 82.74	77.73 ± 13.14; 82.20
FFMI (kg·m^-2^)	22.12 ± 2.87; 21.39	22.65 ± 2.65; 22.00
C	20.26 ± 1.35; 20.78	21.05 ± 1.11; 21.22
T	23.99 ± 2.83; 25.01	24.24 ± 2.86; 25.37
FM (kg)	7.62 ± 2.98; 7.25	8.29 ± 3.18; 8.19^#^
C	5.98 ± 2.37; 5.69	6.58 ± 3.02; 6.29
T	9.27 ± 2.75; 9.31	10.01 ± 2.51; 10.05
FMI (kg·m^-2^)	2.46 ± 0.89; 2.36	2.68 ± 0.99; 2.67^#^
C	2.02 ± 0.80; 1.78	2.22 ± 1.02; 1.96
T	2.90 ± 0.82; 3.01	3.14 ± 0.81; 2.87
PF%	9.88 ± 2.89; 9.32	10.39 ± 3.06; 9.87
C	9.09 ± 3.73; 7.76	9.37 ± 3.66; 8.13
T	10.66 ± 2.17; 10.76	11.41 ± 2.25; 10.07

*differences T from C, ^#^difference Post from Pre.

**Table 2:** Results from the Wingate test for judoists changes during their preparation period (mean ± SD, Median)

	**Pre**	**Post**
RTW (J·kg^-1^)	285.6 ± 17.98; 283.1	283.3 ± 17.4; 286.7
C	294.9 ± 17.42; 296.4	284.1 ± 17.4; 280.8
T	276.3 ± 14.44; 270.4	282.5 ± 19.4; 292.4
RPP (W·kg^-1^)	12.28 ± 0.85; 12.02	12.52 ± 0.59; 12.76
C	12.17 ± 0.88; 12.04	12.12 ± 0.60; 11.98
FI (%)	46.33 ± 6.23; 44.40	44.83 ± 5.63; 44.55
C	43.42 ± 5.31; 43.28	40.99 ± 2.99; 40.39*
T	49.23 ± 6.17; 51.61	48.67 ± 5.06; 46.10
toPP (s)	3.99 ± 0.71; 4.20	3.68 ± 0.77; 3.78^#^
C	4.29 ± 0.28; 4.35	3.94 ± 0.52; 3.81
T	3.69 ± 0.92; 4.01	3.42 ± 0.95; 3.31
tuPP (s)	3.30 ± 0.93; 3.35	3.13 ± 0.55; 3.09
C	3.38 ± 0.64; 3.26	3.30 ± 0.51; 3.41
T	3.22 ± 1.24; 3.44	2.96 ± 0.60; 3.33
La (mmol·l^-1^)	14.35 ± 1.34; 4.31	14.73 ± 1.05; 15.08
C	14.44 ± 1.39; 14.61	14.99 ± 1.15; 15.28
T	14.26 ± 1.44; 14.01	14.47 ± 1.00; 14.25

*differences T from C, ^#^difference Post from Pre.

**Table 3:** Indices which characterize aerobic power in judoists during their preparation period (mean ± SD; Median)

	**Pre**	**Post**
VO_2_max (ml·kg^-1^·min^-1^)	59.04 ± 7.26; 61.1	58.49 ± 5.75; 58.7
C	63.98 ± 2.64; 63.4*	62.80 ± 4.23; 61.8*
T	54.1 ± 7.10; 54.2	54.18 ± 3.16; 53.6
HRmax (bpm)	194.2 ± 10.6; 197	193.8 ± 9.31; 195
C	196.6 ± 8.44; 198	195.8 ± 11.19; 200
T	191.8 ± 12.93; 197	191.8 ± 7.73; 194
HR_TDMA_ (bpm)	167.4 ± 6.04; 166	163.8 ± 11.49; 163
C	168.6 ± 7.83. 170	166.0 ± 2.75; 165
T	166.2 ± 4.15; 165	161.6 ± 11.06; 162
%HRmax (%)	86.37 ± 4.33; 87.1	84.66 ± 6.28; 85.4
C	85.79 ± 2.94; 86.9	84.9 ± 6.35; 85.9
T	86.94 ± 5.72; 87.3	84.42 ± 6.95; 84.8
%VO_2_max (%)	80.58 ± 10.59; 79.2	80.78 ± 6.88; 79.9
C	74.73 ± 5.03; 74.9	76.13 ± 3.48; 75.3*
T	86.43 ± 11.89; 85.6	85.43 ± 6.35; 85.5
La (mmol·l^-1^)	11.65 ± 1.34; 12.0	12.39 ± 1.98; 11.6
C	11.43 ± 1.60; 11.8	10.39 ± 1.52; 12.4
T	11.86 ± 1.16; 12.2	11.39 ± 2.00; 11.2

*differences T from C, ^#^difference Post from Pre.

The results obtained in the SJFT test performed after the six-week training turned out to be better compared to those before training (Table 4). During next segments of the SJFT test, an increase was observed in the number of throws. The differences between the medians were significant for the Throws in Total (2.67, P < 0.01), but not for Index in SJFT (P > 0.05). Mann–Whitney U test in the second measurement revealed no significant differences between medians of groups T and C in BM, indices of body build and composition, whereas in the first measurement a significant difference was noticed only at FFM (Z = 2.09, P < 0.05). The level of total work in Wingate test was significantly different in second measurement (Z = 2.09, P < 0.05), however the difference disappeared when total work was relatively counted (RTW) (P > 0.05). The groups differed in the post-test moment (T > C) in the level of fatigue index FI (Z = 1.98, P < 0.05). No difference was found in aerobic power. The results from specific test (Throws in Total) showed significant differences (T < C) at both, pre- and post-test moments (Z = 2.09, P < 0.05).

**Table 4:** SJFT results and Index in SJFT which characterize special fitness in judoists during their preparation period (mean ± SD, Median)

	**Pre**	**Post**
Segment A (n)	6.0 ± 0.5; 6	6.2 ± 0.6; 6
C	6.2 ± 0.4; 6	6.6 ± 0.5; 7
T	5.8 ± 0.4; 6	5.8 ± 0.4; 6
Segment B (n)	10.7 ± 1.1; 11	11.1 ± 1.0; 11.5
C	11.4 ± 0.5; 11	11.8 ± 0.4; 12
T	10.0 ± 1.0; 10	10.4 ± 0.9; 10
Segment C (n)	10.2 ± 1.4; 10.5	10.6 ± 1.1; 11
C	11.2 ± 0.8; 11*	11.4 ± 0.5; 11*
T	9.2 ± 1.1; 9	9.8 ± 0.8; 10
Throws in Total	26.9 ± 2.7; 27.5	27.9 ± 2.4; 28.5^#^
C	28.8 ± 1.6; 28*	29.6 ± 1.3; 29*
T	25.0 ± 2.1; 25	26.2 ± 1.9; 26
SJFT Index	12.28 ± 1.47; 12.25	12.06 ± 1.22; 12.18
C	11.39 ± 1.24; 12.21*	11.38 ± 1.33; 11.79
T	13.17 ± 1.16; 12.56	12.75 ± 0.63; 12.88

^*^differences T from C; ^#^difference Post from Pre.

## Discussion

For many years, specialists have been seeking for the factors which determine skill level in judoists. Recent studies [22] have demonstrated that, in the opinion of coaches, a technical schooling mostly contributed to sports result (23.4%). Another factors were psychological and tactical preparation (loading 20.1 and 18.0%, respectively). Our longitudinal study was connected with the indices of body build and motor fitness preparation, which contributed to 14.8 and 14.2%, respectively [22]. Franchini et al. [23] and Kubo et al. [24] demonstrated that the competitive success in judo, with an exception of the heaviest weight category, depends on the low fat content in judoists. This suggestion has not been supported by other study [25] which compared exclusively medal winners. There are different ways of calculating percent of fat. One of the methods (Jackson and Pollock formula) develops several formulas based upon a quadratic relation and the function of age groups. Sum of three skinfolds (chest, abdomen and thigh) is used in formula. These three skinfolds were selected by Jackson i Pollock 1978 [26] because of their high intercorrelation with the sum of seven (included subscapula and triceps) and it was thought that they would provide a more feasible field test. The Slaughter et al. [15] formula, which was used in present study, includes two skinfolds measurements (subscapula and triceps) for white postpubescent boys and adults men. During the first and the second measurement in the present study, an increase in body mass was observed, primarily caused by a significant increase in FM. Radovanović et. al. [27] found an increase in body mass as early as after a 2-week training aided with creatine monohydrate. Although mean BMI in our study exceeded 25 kg^.^m^-2^, the percent fat in body mass was not significantly elevated and was typical of the representatives of this sport [28]. Elite judoists had significantly larger fat-free mass than university judo athletes who did not participate in intercollegiate competitions [24]. It is difficult to provide the answer to the question of whether the subjects (this study) reduced body mass during five days before Banska Bystrzyca Tournament by means of reduction in PF or they suffered a loss in fat-free mass, which was observed in elite judoists by Proteau et al. [29]. Nevertheless, comparison of the results of body mass obtained during the measurement 2, in 6 of 7 competitors suggested the necessity of reduction of BM2 from 1 to 6.6 kg (from 1.0% to 9.9%, the mean 4.7±3.0%) in order to meet the requirements of weight category limits.

Reducing weight of some judokas during training period was part of a training procedure, however, not all of the contestants, who participated in the study, was qualified for competition. The judoists, whose weight was above weight category limits, had still 5 days before the beginning of the contest, which is typical time for rapid weight loss of weight-cyclers [30]. Since the fights are carried out within weight categories, body mass control is incorporated in judoists’ training regimes [31], but it does not necessarily have an impact on the reduction in motor abilities because the reduction in BM does not exceed 5% [32]. Kubo et al. [24] suggested that because of the division into the weight categories, the contestants with lower PF should reach higher sport skill level. However, no unequivocal results from studies in this field have been presented so far in the available literature [28].

From the physiological point of view, anaerobic power and capacity, strength, and aerobic power have been considered the main characteristics to be developed by judo players [24,28]. Anaerobic capacity is critical to the effectiveness of techniques used in attack and defense. According to Franchini et al. [33] the anaerobic system provides the short, quick, all-out bursts of maximal power during the match, while the aerobic system contributes to the athlete’s ability to sustain effort for the duration of the combat and to recover during the brief periods of rest or reduced effort. Therefore, we observed the post-training changes in the indices, with the most particular development being the time to obtaining peak power (toPP). Contrary to the placebo group, judo contestants who were supplemented with creatine malate for 6 weeks had significantly higher values of the fatigue index (FI). These results were a natural consequence of faster depletion of muscle phosphagen stores (ATP and CP), mainly in skeletal muscles of type II, caused by generating higher peak power. However, the supplementation with creatine compounds showed no effect on aerobic capacity (VO_2_max), which was consistent with the observations by [4,34]. In the study carried out among track and field athletes, mainly sprinters and long distance runners, these authors did not find significant changes in aerobic capacity after the supplementation with creatine malate.A significant increase, however, was found only in sprinters in peak power (PP and RPP) and total work (TW and RTW). The improved effect of supplementation in athletes in speed and strength sports compared to endurance athletes is likely to be caused by different structure of skeletal muscles. In those with the advantage of fast-twitch fibers of IIa and IIx type, the effectiveness of cytoplasmic aerobic processes is significantly higher than in free cells (of type I) and the creatine in this form can be better absorbed and utilized for the re-synthesis of ATP. Radowanović et al. [27] had subjects use creatine monohydrate and found that, after two weeks, physical capacity in supplemented judo contestants was improved. An anaerobic test focused on upper limbs showed RPP significantly higher than in a placebo group. In the study by [34], the authors did not observe changes in VO_2_max after the supplementation. Moreover, it was found that the level of some physiological indices (VO_2_max and HRmax) was slightly reduced. Very interesting are the differences in threshold levels using the criteria of %VO_2_max. These differences might have practical implications for selection of the aids used in endurance training based on the criterion of anaerobic threshold.

Using the SJFT standards [11], the level of preparation of the study group can be assessed as good (based on Total of Throws and Index in SJFT). Although only two competitors could be assessed as excellent during the first measurement, the second measurement showed five subjects reaching this level. No changes similar to the authors’ study were observed during a two-week experiment [27] focused on the supplementation with creatine monohydrate. In the present study it was the training factor rather than the supplementation which positively affected the results. Lack of differences caused by the supplementation can be explained with almost full correlation (r = 0.99, P < 0.001) between the results from SJFT2 and SJFT1. Only one subject (from the placebo group) did not improve his best result in Throws in Total (n = 31) and his value of the index reduced from 9.48 to 9.11. Serbian researches explained the lack of effect in the SJFT test with its specific nature compared to the laboratory tests [27]. During another experiment, which took 12 weeks, these authors demonstrated a satisfactory improvement in the value of Index in SJFT, regardless of whether the athletes utilized additional endurance training regimes or not. They demonstrated, both in experimental and control groups, the effect of training on RPP level, both during the Wingate test for lower and upper limbs. In the experimental group, who were additionally performing endurance sub-threshold (AnT) exercise, in transition zone and over the AnT threshold, the authors found a significant reduction in PF and BM, and an increase in relative value of VO_2_max during bicycle test for upper and lower limb [35]. Serbian subjects did not show high sport skill level since their Index in SJFT before the experiment and after the experiment ranged from 15.86 (very poor) to 13.24 (average) and did not differ between the groups who used different training regimes. We decided not to compare the findings obtained in our study since it resulted from a different experimental procedure. Russian authors [36] demonstrated that intensification of the training regimes in an Olympic judo team with the exercise of anaerobic character leads to a significant development of special endurance, accompanied by a reduction in aerobic capacity. The judoists training, with fighting bouts of intermittent character, caused integration of anaerobic and aerobic capacity. According to Thomas et al. [10] “judo may be a unique sport in that not only must effort be required equally of upper and lower body, but the training process must require a fine integration between aerobic and anaerobic training.” Based on the evaluation of energy consumption during performing the SJFT test, with its temporal structure and character of movements imitating a fight, the energy cost depends on utilization of the alactic and lactic anaerobic systems and the aerobic system. The alactic energy system presented a higher contribution when compared with both aerobic and lactic energy systems, and the lactic energy system had a lower contribution compared to the aerobic system [37]. Therefore, it seems undisputable that training regimes should periodically incorporate an improvement in a variety of aspects of physical capacity in judoists.

## Conclusions

The multifaceted judo training is conducive to the development of both FM and FMI. Use of supplementation of the diets with creatine malate does not cause an increase in body mass greater than in the control group. Shorter time to obtain peak power toPP is conducive to faster execution of rapid planned actions in attack or defense. Pre and post-training aerobic power did not change so it was not supplementation-dependent. Creatine malate did not affect the results in SJFT. There are many determinants of the judo fight results e.g. technical, tactical, physiological and psychological factors, one of them could be supplementation but it can not be treated as a separate improving factor. The significant improvement in Total Throws in SJFT with the unchanged Index in SJFT suggests better neuromuscular adaptations compared to those occurring in circulatory and respiratory systems. The results obtained during the SJFT test depend not only on energy resources but also on the exercises which improve the technique of performing typical grip-and-throw judo actions, despite the ensuing fatigue.

## Competing interests

The authors declare that they have no competing interests.

## Authors’ contributions

SS was the principle investigator of the study. AKT, KSP, AT, AK aided with data collection and analysis. SS, AKT, KSP, MC, AK and AT conceived parts of the study, and participated in its design and coordination as well as helped to draft the manuscript. MC provided the supplements. All authors read and approved the final manuscript.
